# Supplementary material for: Bacterial DNA promotes Tau aggregation
Source: Sci Rep. 2020 Feb 11;10:2369. doi: 10.1038/s41598-020-59364-x (PMC7012890; doi:10.1038/s41598-020-59364-x)
Supplement: Supplementary file 1 — Supplementary information . [file 41598_2020_59364_MOESM1_ESM.docx]

**Bacterial DNA promotes Tau aggregation**

George Tetz, Michelle Pinho, Sandra Pritzkow, Nicolas Mendez,

Claudio Soto and Victor Tetz

Supplementary table 1

**Assessment of nucleic acid purity**

| **Probe** | **A260/A280 ratio** |
| --- | --- |
| *P. aeruginosa* ATCC 27853 | **1.856** |
| *Porphyromonas gingivalis* | **1.814** |
| *E. coli* ATCC 25922 | **1.904** |
| *Escherichia coli* 472217 | **1.949** |
| *Borrelia burgdorferi* | **1,811** |
| *Tetzerella alzheimeri* VT-16-1752 | **1,897** |
| *Tetzosporium* *hominis* | **1.882** |
| *Candida albicans* | **1.905** |
| Human genomic DNA | **1.964** |
